# Longitudinal Stability of Reading Difficulties: Examining the Effects of Measurement Error, Cut-Offs, and Buffer Zones in Identification

**DOI:** 10.3389/fpsyg.2019.02841

**Published:** 2020-01-10

**Authors:** Maria Psyridou, Asko Tolvanen, Marja-Kristiina Lerkkanen, Anna-Maija Poikkeus, Minna Torppa

**Affiliations:** ^1^Department of Teacher Education, Faculty of Education and Psychology, University of Jyväskylä, Jyväskylä, Finland; ^2^Department of Psychology, Faculty of Education and Psychology, University of Jyväskylä, Jyväskylä, Finland

**Keywords:** reading difficulties, stability, measurement error, cut-offs, simulation, reading fluency, reading comprehension

## Abstract

This study examined the stability of reading difficulties (RD) from grades 2 to 6 and focused on the effects of measurement error and cut-off selection in the identification of RD and its stability with the use of simulations. It addressed methodological limitations of prior studies by (a) applying a model-based simulation analysis to examine the effects of measurement error and cut-offs in the identification of RD, (b) analyzing a non-English and larger sample, and (c) examining RD in both reading fluency and reading comprehension. Reading fluency and reading comprehension of 1,432 Finnish-speaking children were assessed in grades 2 and 6. In addition to the use of single cut-off points on observed data, we used a simulation approach based on an estimated structural equation model (SEM) in order to examine the effect of measurement error on RD identification stability. We also examined the effect of single cut-offs by using a simulation-based buffer zone. Our results showed that measurement error affects the identification of RD over time. The use of a simulation-based buffer zone could control both the effects of measurement error and the arbitrariness of single cut-offs and lead to more accurate classification into RD groups, especially for those with scores close to the cut-offs. However, even after controlling for measurement error and using buffer zones, RD was not stable over time for all children, but both resolving and late-emerging groups existed. The findings suggest that reading development needs to be followed closely beyond the early grades and that reading instruction should be planned according to individual needs at specific time points. There is a clear need for further consideration of the mechanisms underlying the stability and instability of RD.

## Introduction

Studies on reading difficulties (RD) have mainly focused on reading development during the early grades, and long-term longitudinal follow-up studies are scarce. The customary assumption about the persistence of RD is contested by findings in recent studies on the stability of RD. These studies indicate that in addition to persistent RD cases, there are those with resolving RD (i.e., normal reading skills in later grades despite RD being identified during early grades) and those with late-emerging RD (i.e., RD identified during the later grades despite normal early reading skills) (e.g., Catts et al., 2012; Torppa et al., 2015; Etmanskie et al., 2016). These unstable groups are of particular interest because they may provide more understanding on the developmental risk and supportive mechanisms in RD. Such knowledge is useful for more accurate identification of children with RD and for planning support for children and youth with RD. But do these groups truly exist, or are they simply a result of measurement error? When we categorize continuous reading distribution at two time points using arbitrary cut-offs, is it possible that the changes in RD classification are actually due to measurement error?

We cannot answer these questions based on the previous studies on RD stability because of methodological limitations. Such limitations include the use of (arbitrary) cut-offs in reading skill distributions to identify RD cases (Francis et al., 2005; Branum-Martin et al., 2013), not examining the role of measurement error played in the stability of diagnosis across time, small sample sizes (Leach et al., 2003; Lipka et al., 2006), and the use of very lenient 20–25% cut-offs in RD identification (e.g., Lipka et al., 2006; Etmanskie et al., 2016). Furthermore, nearly all studies except for Torppa et al. (2015) have been conducted in English, which constrains the generalization of the findings to other orthographies. Torppa et al. (2015), however, involved only a small number of Finnish children with RD from a specific sample with family risk for dyslexia and examined only reading fluency. Our study examines the effects of measurement error and cut-offs in the longitudinal stability of RD identification across two time points, grades 2 and 6, by using a structural equation model (SEM)-based simulation approach. Examining the effects of measurement error with the use of observed data is not possible because measurement error is constant in observed variables and cannot be manipulated. It can be manipulated though with the use of simulations (Schatschneider et al., 2016). In addition to the use of single cut-off, we examined the stability of RD identification by using a simulation-based buffer zone in order to handle both the effects of the use of single cut-off on continuous distributions (Shankweiler et al., 1999) and the effects of the measurement error. In our study, we used a large-population-based sample of Finnish-speaking children and included both reading fluency and reading comprehension.

### Reading Fluency and Reading Comprehension Difficulties

One of the main aims of education is to teach young children to read and use reading for learning. To be considered a skilled reader, an individual must be able to read accurately and fluently and comprehend the meaning of what was read. Reading accuracy, fluency, and comprehension are closely linked skills, particularly in the early phases of reading development, and reading comprehension can only develop after some basic word identification skills have been achieved (e.g., Florit and Cain, 2011). According to the verbal efficiency theory (Perfetti, 1985), good reading fluency skills facilitate reading comprehension because automaticity in decoding reduces the resource demands of cognitive processes (e.g., memory and attention), which can then be allocated to comprehension. The strength of the relationship between reading fluency and reading comprehension diminishes over time (Florit and Cain, 2011; Torppa et al., 2016; Santos et al., 2019) as children become “fluent enough”. This finding is in accordance with the Simple View of Reading (SVR) model (Gough and Tunmer, 1986; Hoover and Gough, 1990), which states that reading comprehension is a product of two separable abilities – decoding and linguistic comprehension. Several studies over the past 30 years have supported the SVR model (e.g., Catts et al., 2006; Tunmer and Chapman, 2012; Torppa et al., 2016; see García and Cain, 2014, for a meta-analysis). Based on SVR, it is thus expected that, although decoding and reading comprehension are highly related skills, four discrete groups of RD can be identified: only decoding difficulties, only reading comprehension difficulties, both decoding and reading comprehension difficulties, and no RD. Evidence for the dissociated reading skill development in decoding (accuracy and/or fluency) and reading comprehension has been shown repeatedly (e.g., Catts et al., 2003; Torppa et al., 2007; Florit and Cain, 2011).

As reading fluency and reading comprehension are associated, but difficulties with either can emerge; the examination of RD stability should include both. We base our RD stability analysis on a model that includes reading fluency and comprehension, their stability in time, their correlations at both time points, and cross-lagged effects. We do not include reading accuracy in our model because in transparent orthographies (Aro and Wimmer, 2003; Seymour et al., 2003; Aro, 2017) acquisition of high accuracy is a fast process and because almost all children read accurately after 1 year of formal instruction of reading (Lerkkanen et al., 2004; Landerl and Wimmer, 2008; Soodla et al., 2015).

### RD Stability Over Time and the Challenge With Measurement Error in Cut-Off-Based RD Identification

Five studies have addressed the stability of RD classification over time (Leach et al., 2003; Lipka et al., 2006; Catts et al., 2012; Torppa et al., 2015; Etmanskie et al., 2016). Overall, these studies suggest that while some children have stable RD, many also show difficulties only in later grades despite good early reading skills (late-emerging RD). Some studies have also reported that there are individuals whose RD resolve over time (Catts et al., 2012; Torppa et al., 2015). One of the most cited studies of late-emerging poor readers was conducted by Leach et al. (2003), in which 161 grade 4 and grade 5 students were assessed based on school and parental reports. As a cut-off, they used a combination of the research-based estimation that 10–20% of children have RD and the range of reading abilities (including both word reading and reading comprehension measures) of the group with early school-identified and persistent RD. Thirty-one children with late-emerging RD were identified: 11 with word RD, 10 with reading comprehension difficulties, and 10 with both word reading and reading comprehension difficulties. In another longitudinal study, Lipka et al. (2006) examined word reading among 44 children with early RD, of which 22 had RD in grade 4 and 22 were typical readers. Using the 25th percentile as a cut-off in a standardized oral reading test, three subgroups of RD were identified: persistent RD (*N* = 7), borderline RD (*N* = 7), and late-emerging RD (*N* = 8). However, in their studies Leach et al. (2003); Lipka et al. (2006), and Torppa et al. (2015) used single cut-off points on raw variables with measurement error included. Consequently, it is possible to include false-positive and false-negative cases, which can affect the reliability of the results and the prevalence of each group.

Setting a cut-off value in reading ability distribution is a practical tool for identifying RD. However, it causes uncertainty in research findings because of measurement error (Francis et al., 2005; Branum-Martin et al., 2013; Schatschneider et al., 2016). When we use a reading test, there is going to be some measurement error, and as a result, setting a cut-off based on the raw scores will lead to misclassifications. If an individual’s score is slightly above the cut-off, the measurement error could cause his/her observed score to fall below the cut-off, leading him/her to be falsely identified as having RD. Measurement error can thus affect the accurate identification of children with RD. It can also affect the stability of RD, because changes in RD status can reflect either a true change or be due to the effect of measurement error at either or both time points.

The effects of the use of cut-off points on raw variables with measurement error included were tested in the only non-English RD stability study (Torppa et al., 2015). In this Finnish study, the stability of RD status was examined from grades 2 to 8. In the study, 182 children participated, of which 101 had family risk for RD and 81 had no risk. Three reading speed tasks were used for the identification of RD in grades 2 and 8, and the 10th percentile was used as a cut-off point in the distribution of the children without family risk. Four groups were identified: no RD (*N* = 127), late-emerging RD (*N* = 18), resolving RD (*N* = 15), and persistent RD (*N* = 22). In addition to the cut-off-based identification of RD groups, a simulation approach was used to examine how many children would have changed their group due to the unreliability of the measurement. The simulation results showed that 10 out of 33 RD children were misclassified. While the simulation did confirm that RD seem not to be stable for all participants, it also showed that measurement error had a clear effect.

A different methodology was used by Catts et al. (2012), who examined the prevalence of late-emerging RD with a form of latent transition analysis (LTA) in a sample of 493 children followed through grades 2, 4, 8, and 10. Importantly, they examined RD in both word reading and reading comprehension. The LTA provides a good solution for modeling the transitions between latent classes over time. At each time point, four latent classes were allowed in the model: typical reader, word RD, reading comprehension difficulties, and both word reading and reading comprehension difficulties. The cut-off of 1 standard deviation below the weighted sample mean was used for the identification of RD at each time point. In their analysis, six groups were identified across time, two stable over time, and four with transitions. This study controlled measurement error in the LTA mover–stayer model by using multiple binary indicators. Although measurement error was controlled, the actual effects on transitions were not addressed. In addition, although all possible combinations for late-emerging RD were examined (late-emerging word RD, late-emerging reading comprehension difficulties, late-emerging word RD and reading comprehension difficulties), the persistent and resolving classes were not examined.

It is possible though that the single cut-offs contribute to false impressions of the distinctness of the RD groups if many resolving and late-emerging individuals are scoring just above the cut-off value. Shankweiler et al. (1999) argued that using a buffer zone instead of simple cut-off points divides better those with and those without RD. The latest edition of the American Psychiatric Association (2013) *Diagnostic and Statistical Manual of Mental Disorders* (DSM-5) includes severity ratings, which reflect the idea of continuous reading distribution. Developmental disorders are the results of many risk factors and are better seen as dimensional disorders rather than diagnostic categories (Pennington, 2006; Snowling and Hulme, 2012; van Bergen et al., 2014). This is also in line with the notion of the arbitrariness of cut-off points for the classification into learning disorder groups (Moll et al., 2014). Etmanskie et al. (2016) used a buffer zone in their examination of the prevalence and persistence of late-emerging and early identified RD in a sample of 964 children. They used the 25th percentile as a cut-off for the identification of RD (word reading and/or reading comprehension), but to be considered a typical reader, a child’s score needed to be at or above the 35th percentile (a buffer zone between the 25th and 35th percentiles). In grade 4, five groups were identified: typical reading skills (*N* = 694), word RD (*N* = 7), reading comprehension difficulties (*N* = 121), word reading and reading comprehension difficulties (*N* = 24), and borderline reading skills (*N* = 118). The children with poor word reading and/or reading comprehension difficulties were further regrouped into early identified, late-emerging, and inconsistent readers based on their performance in grades 1, 2, and 3.

### The Present Study

The aim of the current study is to examine the effects of measurement error and the effects of single cut-offs in the stability of RD identification from grades 2 to 6. The study aims to address the methodological limitations of the previous studies on RD stability by (a) applying a model-based simulation analysis to examine the effects of measurement error and single cut-offs in the identification of RD, (b) analyzing a non-English and larger sample relative to previous ones, and (c) examining RD in both reading fluency and reading comprehension. The advantages of the use of simulation are threefold: we can examine the effects of measurement error and the effects of single cut-offs on transitions; we can examine all possible combinations for reading fluency and reading comprehension between grades 2 and 6 for all the groups (persistent RD, late-emerging RD, resolving RD); and our larger sample allows us to identify more groups that probably would not have been identified in observed data because some of these groups might not have been big enough. In addition, in our study, we focus on the beginning and the end of primary school in Finland. Because in grade 1 (during which formal instruction of reading begins), it would be difficult to assess accurately reading comprehension, our first assessment point was grade 2. Grade 6 is the last grade of primary school. After that, children enter junior high school and high school where they are taught by subject teachers instead of the classroom teacher.

The research questions of the present study are as follows:

1. How stable are reading fluency and reading comprehension RD from grades 2 to 6?
2. What is the effect of measurement error on the estimation of RD stability over time from grades 2 to 6?
3. What is the effect of using single cut-offs compared with a buffer zone when examining RD stability over time from grades 2 to 6?

We expect that cases of late-emerging and resolving RD would also emerge in the present data (e.g., Leach et al., 2003; Catts et al., 2012; Torppa et al., 2015; Etmanskie et al., 2016). We also anticipated that the simulation approach would reveal an effect of measurement error (Schatschneider et al., 2016) and that the use of a buffer zone would lower the percentages of changing RD groups (resolving and late-emerging).

## Materials and Methods

### Participants and Procedure

The present study is part of the longitudinal First Steps Study (Lerkkanen et al., 2006), a follow-up of approximately 2,000 children from kindergarten to grade 6. The aim of the First Steps Study is to examine the development of children’s learning and motivation in the family and school contexts. The sample was drawn from four municipalities: two in central, one in western, and one in eastern Finland. In three of the municipalities, an invitation was sent through schools to the whole age cohort of children, and in the fourth (urban) municipality, the invitation for participation was sent to approximately half of the age cohort. At the beginning of the study, the children’s parents and teachers were asked for written consent. Of the parents who were contacted, 78–89%, depending on town or municipality, agreed to take part in the study. Of the children’s mothers, 7.6% had no education beyond secondary school, 30.2% had a vocational school degree, 23.8% a vocational college degree, 9.9% a bachelor’s degree, 24% a master’s degree, and 4.6% a doctoral degree. Of the children’s fathers, 7.9% had no education beyond secondary school, 33.2% had a vocational school degree, 23.7% a vocational college degree, 9.9% a bachelor’s degree, 19% a master’s degree, and 6.3% a doctoral degree. Parental education distribution was very close to the national distribution of Finland (Statistics Finland, 2007). The sample was highly homogeneous in ethnic and cultural background (e.g., Finnish-speaking schools and students). The study was approved by the Ethical Committee of the University of Jyväskylä and at the beginning of the study the children’s parents, and teachers provided informed written consents for participation. During the study, also the children gave their written consent to participate.

The present study involved assessments at two time points – the end of grade 2 (Spring 2009) (8 years) and the end of grade 6 (Spring 2013) (13 years). Only children (*N* = 1,432; 662 girls and 770 boys) for whom data were available for both the grade 2 and grade 6 assessments were included in the analyses. All participants who were assessed in grade 6 were included in the current sample. In grade 6 spring, 1,824 12- to 13-year-old children participated: 863 girls (47.31%) and 961 (52.69%) boys. Of them, 1,458 participated also in grade 2 spring (8–9 years old): 680 girls (46.64%) and 778 (53.36%) boys. The sample size of the First Steps Study changed somewhat each year due to factors as shifts in teaching groups or absences during the testing days. In the present study, data from 72 schools and 147 classrooms were used. The SEM described below for the development of reading fluency and reading comprehension from grades 2 to 6 was also constructed using full grade 6 data. There were only minor differences to some of the path estimates ranging from 0.01 to 0.05.

Conducting such a long-term follow-up study is challenging, and some changes in the sample from one assessment time point to another are inevitable. We conducted a missing value analysis in order to examine if missingness was random for the data we used at the current study (*z*-scores for reading fluency composite and reading comprehension in grades 2 and 6). We used the Little’s (1988) tests of missing completely at random (MCAR), which showed that the data were not MCAR, χ^2^(14) = 62.29, *p* < 0.001. Reading fluency composite score and reading comprehension in grade 2 had 20.34 and 21.27% of the cases missing. Reading fluency composite score and reading comprehension in grade 6 had 0.22 and 0.16% of the cases missing. The one-way ANOVA analysis comparing the reading fluency and reading comprehension performance of the sample included in this study (only those who were assessed in both grades 2 and 6; *N* = 1,432) and the whole sample (*N* = 1,824) showed that those who participated in both grades were somewhat better readers than those who participated only in grade 6 [for reading fluency: *F*(1,1,812) = 47.08, *p* < 0.001; for reading comprehension: *F*(1,1,819) = 7.46, *p* < 0.01]. However, the effect sizes were small for reading fluency (*d* = −0.39) and negligible for reading comprehension (*d* = −0.15).

The comprehensive education, grades 1–9, starts at the fall from the year in which the child turns 7 years of age, which is rather late compared with other countries. Before entrance to elementary school, all 6-year-olds attend 1-year kindergarten education. One goal of kindergarten education is to arouse children’s interest in texts and reading and to support emerging pre-reading skills, instead of a systematic instruction of decoding (Lerkkanen, 2018). However, children are read to, and they are also encouraged to play with letters, phonemes, and words (Lerkkanen, 2019). Reading instruction begins at grade 1, and it is based on grapheme–phoneme correspondence (phonics) and a highly transparent Finnish orthography, which makes reading acquisition relatively easy and quick for children (Lerkkanen, 2007). At the end of kindergarten, around 30% of the children can read fluently, around 30% can decode easy words while around 30% of the children show no sign of reading (Lerkkanen et al., 2010; Soodla et al., 2015). Largely due to the consistent nature of the highly transparent orthography of the Finnish language (Aro, 2017), reading accuracy hits a ceiling after a few months of formal reading instruction in grade 1 (Lerkkanen et al., 2004), and basically all children can read accurately by the end of the first school year (Soodla et al., 2015). However, even a highly consistent orthography does not guarantee efficient reading acquisition for all children. RD are typically identified for approximately 5–20% of children in either reading fluency or comprehension, depending on the criteria (Lerkkanen et al., 2010).

In basic education, children do not need to have an official diagnosis in order to have access to special educational services. Teachers and parents along with the students assess the need for extra support (Björn et al., 2016). The most common form of special educational services is the part-time special education provided by a special education teacher (Statistics Finland, 2005). In this form of special education, students study in general education classes and receive support 1–2 h/week from a special education teacher. This kind of support focuses on reading, spelling, and math difficulties. It is implemented in small groups (typically three to four students) or individually if the student faces long-lasting or more severe difficulties or if the student faces difficulties in more than one learning areas (Holopainen et al., 2018).

### Measures

#### Reading Fluency

There were three group-administered tests for the assessment of reading fluency: a word reading fluency task, a word-chain task, and a sentence reading task. Cronbach’s alpha reliability coefficient for the fluency composite was 0.79 in grade 2 and 0.77 in grade 6.

##### Word reading fluency task

The word reading fluency task is a subtest of the nationally normed reading test battery [ALLU–Ala-asteen lukutesti (ALLU–Reading Test for Primary School); Lindeman, 2000]. Each of the 80 items consisted of a picture with four phonologically similar words attached to it. The child silently read the four words and then drew a line to connect the picture with the word, semantically matching it. The words and pictures were frequently used words familiar to young children. For example, there was a picture of a bunny (pupu in Finnish) and the correct word along with three distractors (English word is in parentheses): pipo (cap), papu (bean), and apu (help). Completing the test requires fluent decoding. The score was the number of correct answers within a 2-min time limit. Because of the nature of this timed test, the score reflects both the child’s fluency in reading the stimulus words and accuracy in making the correct choice from among the alternatives. According to the test manual (Lindeman, 1998), the Kuder–Richardson reliability was 0.82 in grade 2 and 0.97 in grade 6.

##### Word-chain task

The word-chain task (Nevala and Lyytinen, 2000) is a timed test with 10 rows of word chains comprising four to six words written together without spaces. The child silently read the words in the chains and, while reading them, indicated the word boundaries by drawing a division line between words. The score was the number of correct responses (maximum 40) within the time limit (1 min 25 s in grade 2). In our sample, the Pearson correlation coefficient between grades 2 and 6 was 0.52.

##### Sentence reading task

The Test of Silent Reading Efficiency and Comprehension (TOSREC; Wagner et al., 2009; Finnish version by Lerkkanen and Poikkeus, 2009) was used to assess silent reading efficiency in grade 2. Respondents were given 3 min to read 60 sentences and verify the truthfulness of as many sentences as possible. In grade 6, a similar task was used, the Salzburg Sentence Reading Test (Landerl et al., 1997, translated into Finnish by Sini Huemer; Pichler and Wimmer, 2006). Respondents were given 2 min to read 69 sentences and verify the truthfulness of as many sentences as possible. The sum score was based on the number of correct items. In our sample, the Pearson correlation coefficient between grades 2 and 6 was 0.67.

#### Reading Comprehension

A group-administered subtest of a nationally normed reading test battery (ALLU; Lindeman, 2000) was used to assess reading comprehension. The children silently read a fiction story and then answered 11 multiple-choice questions and one question in which they had to arrange five statements in the correct sequence based on information gathered from the text. The text contained 114 words in grade 2. The child received 1 point for each correct answer (max = 12). Each child completed the task at his or her own pace, but the maximum time allotted was 45 min. Lindeman (2000) reported Kuder–Richardson reliability coefficients of 0.80 in grade 2 and 0.74 in grade 6.

### Analysis Description

First, we identified the RD groups using cut-off points on the observed data in a similar fashion as previous RD stability studies. We first calculated *z*-scores for reading comprehension and *z*-scores for the three reading fluency tasks in grades 2 and 6 separately. Based on the *z*-scores for reading fluency, we calculated mean composite scores for each grade. We used the 10th percentile as the cut-off value and dichotomized the reading fluency and reading comprehension *z*-score variables accordingly. The four variables were coded at each time point as 0 = typical reader (above the 10th percentile) and 1 = RD (below the 10th percentile) for each case (Table 3).

**FIGURE 1:**
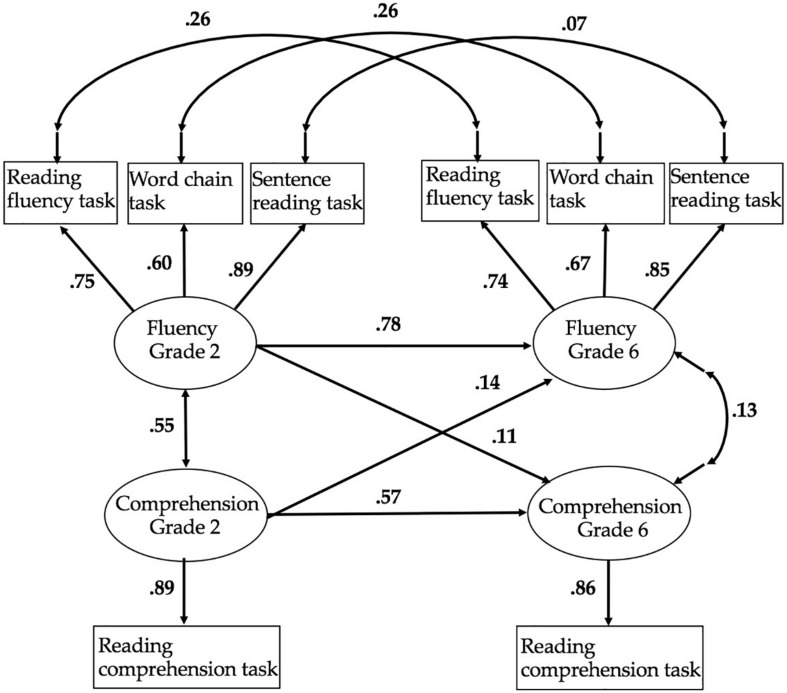
Standardized path estimates of the cross-lagged model. The figure presents standardized estimates using listwise analysis.

The simulation analysis on the effects of measurement error on the RD grouping started by building a SEM for the development of reading fluency and reading comprehension from grades 2 to 6 (see Supplementary Appendix A). The model was constructed using four latent variables consisting of separate factors for reading fluency and reading comprehension in grades 2 and 6 (see Figure 1). The use of latent variables for reading fluency (composed of three measures) and reading comprehension provide reading measures that do not include measurement error. Because reading comprehension had only one measure at each time point, we calculated the correction of attenuation using the Kuder–Richardson reliability estimates for reading comprehension in each grade from the test manual (Lindeman, 2000). In this way, we can set measurement error also in reading comprehension. The model included the stability paths within the reading constructs, the cross-lagged paths between reading fluency and comprehension across time, and correlations between reading fluency and comprehension at both time points.

The structural equation model analyses were carried out using Mplus 7.4 software. Maximum likelihood estimation with robust standard errors (MLR) was used for the analysis. To evaluate model fit, chi-square values and a set of fit indexes were used as follows: (a) the comparative fit index (CFI); (b) Tucker–Lewis index (TLI); and (c) the root-mean-square error of approximation (RMSEA). Good model fit is indicated by a small, preferably non-significant χ^2^, CFI > 0.95, TLI > 0.95, and RMSEA < 0.06 (Hu and Bentler, 1999). Since the chi-square test depends on sample size and is sensitive to a large sample size, the chi-square statistics were not regarded as conclusive.

Next, we estimated model parameters of the SEM model to produce two simulated datasets with 200,000 cases. The first dataset was simulated using parameters related to latent factors, hence corresponding to true scores without measurement error (see Supplementary Appendix B). The second dataset used all the parameters in the model to produce data that include also the measurement error (see Supplementary Appendix C). In this dataset, the error covariances were also included in the simulation equations to correspond to the observed situation. The simulations were produced using the Statistical Package for the Social Sciences (SPSS). We used the estimates for the stability paths, the cross-lagged paths, and the correlations between reading fluency and reading comprehension variables. For each simulation, we entered four discrete variables into the model: fluency in grade 2, fluency in grade 6, comprehension in grade 2, and comprehension in grade 6. The scores for each of the four variables were coded at each time point as 0 = typical reader (highest 90%) and 1 = RD (lowest 10%) for each case. Next, we calculated the frequencies of RD groups in each simulated dataset. By comparing the percentages of RD groups in the simulated samples without and with measurement error, we can examine the effect of measurement error on RD identification and RD identification stability.

The final step of our analysis was to examine RD stability with the use of a buffer zone. We used the same procedure described above, but the four discrete variables that were entered into the model were coded at each time point as 0 = typical reader (highest 75%), 1 = RD (lowest 10%), and 2 = borderline score (lowest 10–25%) for each case. Those with scores in the lowest 10% of the reading fluency and/or reading comprehension distribution were identified as manifesting reading fluency and/or reading comprehension difficulties. Those with scores in the lowest 10–25% range were identified as borderline readers, and those with scores above 25% were identified as having no RD (Tables 7, 8). In this analysis, we focused specifically on the late-emerging and the resolving RD in order to examine how far the scores are from the cut-off and how distinct the two groups are that were identified using the single cut-off.

## Results

### Descriptive Statistics

See Table 1 for descriptive statistics and Table 2 for the correlations between reading fluency and reading comprehension measures in grades 2 and 6. For the reading measures, the stability correlations between grades 2 and 6 ranged from moderate to high (0.49–0.67).

**TABLE 1:** Descriptive statistics for reading fluency and reading comprehension measures in grades 2 and 6.

	***N***	**Minimum**	**Maximum**	**Mean**	**SD**	**Skewness**	**Kurtosis**
**Grade 2**							
Word reading fluency task	1,458	3	58	24.75	7.39	0.46	0.11
Word-chain task	1,458	0	35	11.59	5.93	0.61	0.31
Sentence reading task	1,453	3	60	30.57	8.15	0.02	0.26
Reading comprehension	1,436	0	12	8.73	2.58	–0.79	–0.14
**Grade 6**							
Word reading fluency task	1,820	10	80	47.22	10.94	0.00	0.00
Word-chain task	1,820	1	40	21.63	7.44	0.09	–0.36
Sentence reading task	1,822	4	62	30.61	7.38	0.15	0.28
Reading comprehension	1,821	0	12	7.15	2.55	–0.20	–0.59

**TABLE 2:** Correlations between reading fluency and reading comprehension measures.

	**1**	**2**	**3**	**4**	**5**	**6**	**7**	**8**
**Grade 2**								
(1) Word reading fluency task	1							
(2) Word-chain task	0.48	1						
(3) Sentence reading task	0.66	0.53	1					
(4) Reading comprehension	0.31	0.40	0.43	1				
**Grade 6**								
(5) Word reading fluency task	0.58	0.38	0.56	0.31	1			
(6) Word-chain task	0.43	0.52	0.53	0.39	0.52	1		
(7) Sentence reading task	0.53	0.44	0.67	0.45	0.63	0.52	1	
(8) Reading comprehension	0.23	0.29	0.33	0.49	0.22	0.32	0.38	1

### Identification of the RD Groups With Observed Data

First, RD groups in reading fluency and reading comprehension in grades 2 and 6 were calculated from observed data using the 10th percentile as the cut-off value (Table 3). There was a statistically significant association between RD in grade 2 and RD in grade 6 [χ^2^(9) = 441.71, *p* < 0.001].

**TABLE 3:** Number and percentage of children in each group based on the observed data for those who were assessed in both grades 2 and 6 using the 10th percentile as a cut-off point.

			**Grade 6**				
			
			**RF only**	**RC only**	**RF + RC**	**no RD**	**Total**
Grade 2	RF^a^ only	Number of children	35	17	9	43	104
		% within grade 2	33.65	16.35	8.65	41.35	100.00
		% of total	2.44	1.19	0.63	3.00	7.26
		Adjusted residual	11.58	1.26	3.05	−9.20	
	RC^b^ only	Number of children	7	30	13	38	88
		% within grade 2	7.95	34.09	14.77	43.18	100.00
		% of total	0.49	2.09	0.91	2.65	6.15
		Adjusted residual	0.54	6.36	6.05	−7.99	
	RF + RC	Number of children	12	2	14	11	39
		% within grade 2	30.77	5.13	35.90	28.21	100.00
		% of total	0.84	0.14	0.98	0.77	2.72
		Adjusted residual	6.19	−1.40	11.31	−7.50	
	no RD^c^	Number of children	40	129	13	1,019	1,201
		% within grade 2	3.33	10.74	1.08	84.85	100.00
		% of total	2.79	9.01	0.91	71.16	83.87
		Adjusted residual	−11.27	−4.42	−11.10	15.03	
Total		Count	94	178	49	1,111	1,432
		% within grade 2	6.56	12.43	3.42	77.58	100.00
		% of total	6.56	12.43	3.42	77.58	100.00

*^*a*^RF, reading fluency; ^*b*^RC, reading comprehension; ^*c*^no RD, no reading difficulties.*

Approximately 10% of the participants had persistent RD, most of whom had persistent single reading fluency or reading comprehension difficulties. Most of those with either reading fluency or reading comprehension difficulties in grade 2 continued to have the same difficulty in grade 6, or they developed both reading fluency and comprehension difficulties in grade 6. Most of those with both reading fluency and reading comprehension difficulties in grade 2 continued to have only reading fluency difficulties or both reading fluency and reading comprehension difficulties in grade 6. It was rare for those struggling with any type of RD in grade 2 to move to no RD group in grade 6 and the other way around.

The percentage of children with no RD in grade 2 but some type of RD in grade 6 (late-emerging cases) was quite high (12.71%), and two-thirds of these cases had late-emerging reading comprehension difficulties. Only 2.79% of the children had only late-emerging reading fluency difficulties. The percentage of resolving difficulties (some kind of RD in grade 2 but no RD in grade 6) was smaller (6.42%), and the prevalence of resolving reading fluency and resolving reading comprehension difficulties was quite similar. Both resolving and late-emerging profiles were rare among children with both reading fluency RD and comprehension RD.

In order to describe the severity of RD in each group, we calculated means and standard deviations of the RD groups in reading fluency and reading comprehension in grades 2 and 6 (Table 4). Those with persistent RD performed 1.5 or more standard deviations below the average level in either fluency, comprehension, or both. Similarly, those with resolving RD performed 1.5 or more standard deviations below the average in grade 2, and those with late-emerging RD performed 1.5 or more standard deviations below the average in grade 6. However, in the resolving RD groups, the grade 6 skill performance was still below average, particularly for reading fluency (as low as −0.88 in the combined group). Similarly, the grade 2 reading levels of late-emerging RD groups were also somewhat below average (as low as −0.76 in the combined group).

**TABLE 4:** Descriptive statistics for the groups based on the observed data.

	***N***	**Reading fluency, grade 2^z^**		**Reading comprehension, grade 2^z^**		**Reading fluency, grade 6^z^**		**Reading comprehension, grade 6^z^**	
					
		**Mean**	**SD**	**Mean**	**SD**	**Mean**	**SD**	**Mean**	**SD**
**Persistent**									
RC^a^ → RC	30	–0.37	0.60	–2.15	0.31	–0.37	0.70	–1.58	0.35
RC → RF^b^	7	–0.78	0.41	–1.99	0.30	–1.49	0.16	–0.71	0.31
RC → RF + RC	13	–0.85	0.24	–1.92	0.17	–1.66	0.21	–1.60	0.35
RF → RC	17	–1.47	0.19	–0.78	0.64	–0.63	0.34	–1.54	0.33
RF → RF	35	–1.58	0.33	–0.34	0.66	–1.75	0.40	–0.14	0.78
RF → RF + RC	9	–1.81	0.40	–0.67	0.64	–2.04	0.38	–1.74	0.38
RF + RC → RC	2	–1.66	0.38	–1.83	0.00	–1.19	0.10	–1.66	0.00
RF + RC → RF	12	–1.95	0.36	–2.37	0.42	–1.89	0.41	–0.29	0.56
RF + RC → RF + RC	14	–1.88	0.46	–2.24	0.44	–1.95	0.50	–1.88	0.40
**Late emerging**									
no RD → RC	129	–0.06	0.75	–0.21	0.79	–0.05	0.67	–1.54	0.36
no RD → RF	40	–0.64	0.47	–0.28	0.77	–1.56	0.19	–0.01	0.63
no RD → RF + RC	13	–0.76	0.32	–0.70	0.46	–1.60	0.22	–1.72	0.39
**Resolving**									
RC → no RD^*c*^	38	–0.28	0.54	–2.07	0.33	–0.24	0.63	–0.28	0.57
RF → no RD	43	–1.49	0.24	–0.22	0.74	–0.69	0.48	0.09	0.73
RF + RC → no RD	11	–1.55	0.22	–2.14	0.38	–0.88	0.27	–0.34	0.59
**No RD**									
no RD → no RD	1, 019	0.31	0.88	0.35	0.72	0.32	0.86	0.38	0.76
Total	1, 432								

*Subscript z refers to standardized score. The reading fluency measures are composite scores calculated as sum scores of the standardized scores for the three reading fluency tasks. ^*a*^RC, reading comprehension; ^*b*^RF, reading fluency; ^*c*^no RD, no reading difficulties. On the left side of the arrow is the reading difficulty status in grade 2 [only reading fluency difficulties (RF), only reading comprehension difficulties (RC), both reading fluency and reading comprehension difficulties (RF + RC), or no reading difficulties (no RD)]. On the right side of the arrow is the reading difficulty status in grade 6. Reading fluency and/or reading comprehension difficulties were calculated using the 10th percentile cut-off value.*

Gender was unevenly distributed in the groups, χ^2^(3) = 15.32, *p* < 0.05: there were less boys than expected in the group with no RD in both grades (68.38% of boys compared with 77.80% of girls with adjusted standardized residual = −3.43) (Table 5). In the RD groups, there were no significant differences. Of the boys, 11.55% belonged to the persistent RD groups, 14.39% to the late-emerging group, and 5.68% to the resolving group with adjusted standardized residuals of 2.69, 2.50, and −0.29, respectively. Among the girls, 6.72% belonged to the persistent RD groups, 9.37% to the late-emerging groups, and 6.11% to the resolving groups.

**TABLE 5:** Prevalence of boys and girls in each group based on observed data.

**Group**		**Gender**		**Total**
		**Male**	**Female**	
Persistent	Count	65	33	98
	% with the groups	66.33	33.67	100
	% within gender	11.55	6.72	9.30
	% of total	6.17	3.13	9.30
	Adjusted residual	2.69	−2.69	
Late emerging	Count	81	46	127
	% with the groups	63.78	36.22	100
	% within gender	14.39	9.37	12.05
	% of total	7.69	4.36	12.05
	Adjusted residual	2.50	−2.50	
Resolving	Count	32	30	62
	% with the groups	51.61	48.39	100
	% within gender	5.68	6.11	5.88
	% of total	3.04	2.85	5.88
	Adjusted residual	−0.29	0.29	
no RD	Count	385	382	767
	% with the groups	50.20	49.80	100
	% within gender	68.38	77.80	72.77
	% of total	36.53	36.24	72.77
	Adjusted residual	−3.43	3.43	
Total	Count	563	491	1,054
	% with the groups	53.42	46.58	100
	% within gender	100	100	100
	% of total	53.42	46.58	100

### Simulation-Based Identification of the RD Groups: The Effect of Measurement Error

In order to examine whether measurement error affects the identification and stability of RD across time, we produced one simulation without and another with measurement error.

#### SEM Model

Latent factors for reading fluency and reading comprehension were built in grades 2 and 6 (see Figure 1). In addition to the regression paths across time (grades 2 and 6) and across constructs (reading fluency and reading comprehension), residual covariances for each measure of reading fluency across time were added to the model based on the inspection of modification indices. The model showed good fit with the data, χ^2^(14) = 117.163, *p* < 0.001, RMSEA = 0.072, CFI = 0.977, TLI = 0.954, standardized root mean square (SRMR) = 0.032. The model indicated that reading fluency was very stable across time while reading comprehension was less stable. Of grade 6 reading fluency variance, 60.8% was explained by grade 2 reading fluency and an additional 2% by grade 2 reading comprehension. Of grade 6 reading comprehension variance, 32.5% was explained by grade 2 reading comprehension and an additional 1.2% by grade 2 reading fluency.

Next, based on the model, we produced a simulated dataset without measurement error. We simulated 200,000 cases and identified cases with RD as the lowest 10% of the reading fluency and/or reading comprehension distribution (Table 6). In the simulated data without measurement error, 86.45% were in the stable groups (76.52% no RD; 9.93% persistent RD) and 13.55% demonstrated instability in RD across grades. Of the cases demonstrating instability, 6.50% had resolving RD and 7.05% were identified as manifesting late-emerging RD; most of these manifested late-emerging reading comprehension difficulties.

**TABLE 6:** Percentages and number of individuals in reading difficulty (RD) groups identified with the use of a single cut-off.

**Group**	**Subgroup**	**Simulation without measurement error (group %)**	**Simulation with measurement error (group %)**
Persistent	RC^a^ → RC	1.92 (27)	1.57 (22)
	RC → RF^b^	0.54 (8)	0.73 (10)
	RC → RF + RC	0.47 (7)	0.44 (6)
	RF → RC	0.33 (5)	0.54 (8)
	RF → RF	2.93 (42)	2.56 (37)
	RF → RF + RC	0.68 (10)	0.61 (9)
	RF + RC → RC	0.36 (5)	0.35 (5)
	RF + RC → RF	1.28 (18)	1.02 (15)
	RF + RC → RF + RC	1.42 (20)	0.79 (11)
Persistent total		9.93 (142)	8.61 (123)
Late emerging	no RD^c^ → RC	4.38 (63)	5.10 (73)
	no RD → RF	2.23 (32)	3.26 (47)
	no RD → RF + RC	0.44 (6)	0.60 (9)
Late-emerging total		7.05 (101)	8.96 (129)
Resolving	RC → no RD	3.50 (50)	4.38 (63)
	RF → no RD	2.48 (36)	3.41 (49)
	RF + RC → no RD	0.52 (8)	0.73 (10)
Resolving total		6.50 (94)	8.52 (122)
no RD	no RD	76.52(1,095)	73.92(1,058)

*^*a*^RC, reading comprehension, ^*b*^RF, reading fluency, ^*c*^no RD, no reading difficulties. The number in parentheses shows the number of individuals based on the percentage for *N* = 1,432. On the left side of the arrow is the RD status in grade 2 [only reading fluency difficulties (RF), only reading comprehension difficulties (RC), both reading fluency and reading comprehension difficulties (RF + RC), or no reading difficulties (no RD)]. On the right side of the arrow is the RD status in grade 6. Reading fluency and/or reading comprehension difficulties were calculated using the lowest 10%.*

Next, we produced a simulated dataset with 200,000 cases with measurement error. Overall, 82.53% of the cases were in stable groups from grades 2 to 6 (73.92% no RD; 8.61% persistent RD) (Table 6). The remaining 17.48% of the cases demonstrated instability in RD across grades. Of the cases demonstrating instability, 8.52% had resolving RD, and 8.96% were identified as manifesting late-emerging RD; most of these manifested difficulties in reading comprehension.

Finally, the group sizes produced by the two simulations were compared in order to examine the effects of measurement error. For the simulation without measurement error, the percentage of the stable groups was slightly higher, and the percentage of the cases with instability in RD was somewhat lower. The results, thus, suggest that 1.32% of the children would have not been identified as having persistent RD due to the inclusion of measurement error in the analysis. In other words, in the observed data of 1,432 children, approximately 19 children would be wrongly classified. Similarly, 3.93% of the children would have changed groups due to the inclusion of measurement error in the analysis, which means that in the observed data, 56 out of 1,432 children would be wrongly classified. More specifically, 28 children (1.91%) would have been misclassified as having late-emerging RD and 28 children (2.02%) misclassified as having resolved their RD.

### The Effect of the Use of a Single Cut-Off

The final step of our analysis was to examine RD stability with the use of a buffer zone. In this analysis, we focused particularly on the late-emerging RD (Table 7) and resolving RD groups (Table 8). Similar to the use of a single cut-off, we produced two simulated datasets, one without and one with measurement error. We simulated 200,000 cases for each dataset and identified RD for those cases located in the lowest 10% of the reading fluency and/or reading comprehension distribution. Cases with scores in the lowest 10–25% range were identified as having borderline scores, and cases with scores above 25% were identified as having no RD.

**TABLE 7:** Percentage and number of individuals in late-emerging groups identified with the use of a buffer zone (bz).

**Late-emerging groups identified using a single cut-off**	**Late-emerging groups identified using a buffer zone**	**Simulation without measurement error, group % (*N*)**	**Simulation with measurement error, group % (*N*)**
no RD → RC	no RD → RC	1.69 (24)	2.30 (33)
	no RD → RF(bz) + RC	0.28 (4)	0.42 (6)
	RC(bz) → RC	1.10 (16)	0.98 (14)
	RC(bz) → RF(bz) + RC	0.29 (4)	0.32 (5)
	RF(bz) → RC	0.20 (3)	0.36 (5)
	RF(bz) → RF(bz) + RC	0.28 (4)	0.25 (4)
	RF(bz) + RC(bz) → RC	0.21 (3)	0.24 (3)
	RF(bz) + RC(bz) → RF(bz) + RC	0.33 (5)	0.22 (3)
		**4.38 (63)**	**5.09 (73)**
no RD → RF	no RD → RF	0.29 (4)	0.85 (12)
	no RD → RF + RC(bz)	0.09 (1)	0.22 (3)
	RC(bz) → RF	0.09 (1)	0.26 (4)
	RC(bz) → RF + RC(bz)	0.06 (1)	0.12 (2)
	RF(bz) → RF	0.82 (12)	0.92 (13)
	RF(bz) → RF + RC(bz)	0.26 (4)	0.31 (5)
	RF(bz) + RC(bz) → RF	0.37 (5)	0.37 (5)
	RF(bz) + RC(bz) → RF + RC(bz)	0.25 (4)	0.20 (3)
		**2.23 (32)**	**3.25 (47)**
no RD → RF + RC	no RD → RF + RC	0.04 (0)	0.13 (2)
	RC(bz) → RF + RC	0.06 (1)	0.11 (1)
	RF(bz) → RF + RC	0.14 (2)	0.18 (3)
	RF(bz) + RC(bz) → RF + RC	0.20 (3)	0.18 (3)
		**0.44 (6)**	**0.60 (9)**

*The number in parentheses shows the number of individuals based on the percentage for *N* = 1,432. The bold values show the total number of late-emerging cases.*

**TABLE 8:** Percentages and number of individuals of the resolving groups identified with the use of a buffer zone.

**Resolving groups identified using a single cut-off**	**Resolving groups identified using a buffer zone**	**Simulation without measurement error (ME), group % (*N*)**	**Simulation with ME, group % (*N*)**
RC → no RD	RC → no RD	1.04 (15)	1.65 (24)
	RC → RC(bz)	0.77 (11)	0.82 (12)
	RC → RF(bz)	0.25 (4)	0.41 (6)
	RC → RF(bz) + RC(bz)	0.25 (4)	0.26 (4)
	RF(bz) + RC → no RD	0.27 (4)	0.42 (6)
	RF(bz) + RC → RC(bz)	0.21 (3)	0.24 (3)
	RF(bz) + RC → RF(bz)	0.35 (5)	0.36 (5)
	RF(bz) + RC → RF(bz) + RC(bz)	0.35 (5)	0.22 (3)
		**3.49 (50)**	**4.38 (63)**
RF → no RD	RF → no RD	0.39 (6)	0.93 (13)
	RF → RC(bz)	0.07 (1)	0.19 (3)
	RF → RF(bz)	0.97 (14)	1.06 (15)
	RF → RF(bz) + RC(bz)	0.24 (3)	0.26 (4)
	RF + RC(bz) → no RD	0.11 (2)	0.26 (4)
	RF + RC(bz) → RC(bz)	0.06 (1)	0.11 (2)
	RF + RC(bz) → RF(bz)	0.41 (6)	0.42 (6)
	RF + RC(bz) → RF(bz) + RC(bz)	0.22 (3)	0.19 (3)
		**2.47 (36)**	**3.42 (49)**
RF + RC → no RD	RF + RC → no RD	0.05 (1)	0.15 (2)
	RF + RC → RC(bz)	0.04 (1)	0.10 (1)
	RF + RC → RF(bz)	0.22 (3)	0.28 (4)
	RF + RC → RF(bz) + RC(bz)	0.21 (3)	0.20 (3)
		**0.52 (8)**	**0.73 (10)**

*The number in parentheses shows the number of individuals based on the percentage for *N* = 1,432. The bold values show the total number of resolving cases.*

The results from the use of the buffer zone suggest that most of the cases from the late-emerging and resolving RD groups actually land in the buffer zone. For the late-emerging group, the simulation without measurement error and with the buffer zone showed that only 33 cases would be identified as having late-emerging RD compared with the 101 that were identified with the use of a single cut-off. Of the 33 cases, 28 were identified with late-emerging reading comprehension difficulties and five with late-emerging reading fluency difficulties (Table 7). For the resolving group, the simulation without measurement error and with the buffer zone showed that 28 cases would be identified as manifesting resolving RD compared with the 94 identified with the use of a single cut-off. Of the 28 cases, 19 would be identified with resolving reading comprehension difficulties, 8 with resolving reading fluency difficulties, and 1 with resolving reading fluency and comprehension difficulties (Table 8).

## Discussion

The main focus of the present study was to examine the longitudinal stability of RD identification across two time points, grades 2 and 6 including both reading fluency and reading comprehension. We examined whether RD identification was stable over time even if we control for measurement error and the use of single cut-offs. Our results showed that for some children RD are not stable but also revealed a clear effect of measurement error and the selection of the cut-off in the identification of RD. The findings highlight that measurement error affects the accurate identification of children with RD by causing misclassifications and that the simplicity of the single cut-offs can contribute to false impressions on instability of RD over time.

All the previous studies, except the study conducted by Catts et al. (2012), examining the stability of RD identification, used cut-off points on raw variables with measurement error included (Leach et al., 2003; Lipka et al., 2006; Torppa et al., 2015; Etmanskie et al., 2016). This means that conclusions may be biased by false-positive or false-negative cases due to measurement error causing misclassifications. In this study, we used simulations with models that do and do not include measurement error to estimate the magnitude of this problem. Our findings comparing the results of the simulations with and without measurement error showed the impact of measurement error but still aligned with prior findings suggesting RD instability for a group of children (Leach et al., 2003; Lipka et al., 2006; Catts et al., 2012; Torppa et al., 2015; Etmanskie et al., 2016). In our study, 74–77% of participants were typical readers across time, and each RD group consisted of 7–10% of the participants (depending on the group and the model). Most of the participants (86.45% in the simulation without measurement error and 82.53% in the simulation with measurement error) demonstrated stability in their RD status (no RD and persistent RD). The simulation without measurement error revealed, however, larger proportions of the stable groups (persistent RD and no RD) and smaller proportions of late-emerging and resolving RD compared with the simulation with measurement error. These differences were expected because the results from the simulation with measurement error include false-positive or false-negative cases because of the effect measurement error, which affects the reliability of the estimation of the prevalence of each RD group. Although the differences in the prevalence of the groups were small, they show that measurement error has an effect on the longitudinal stability of RD identification. Overall, though, the findings supported that the unstable groups exist, even if we control for measurement error, which has been a problem in most previous studies.

Catts et al. (2012) study is the only previous study comparable to the present simulation analysis as they used LTA, which relied on multiple indicators for each reading class, and their findings were thus less affected by measurement error. Our results from the analyses using single cut-offs were in line with Catts et al. (2012) in that there was a higher prevalence of late-emerging reading comprehension than late-emerging reading fluency difficulties, but the findings show differences in the proportion of children in each RD group. More specifically, the present study identified a smaller proportion of late-emerging cases (7.05% compared with 13.40%) and more resolving cases (6.50% compared with 1.90%). Of the children with RD in the Catts et al. (2012) study, 42% had late-emerging RD and 6% had resolving RD; in our study, the percentages were 30 and 28%, respectively.

It is possible that differences in orthography, in assessment ages of children, or differences in the criteria used for the identification of RD, explain the differences between the present study and that by Catts et al. (2012). Finnish orthography is highly transparent, with one-on-one correspondence between phonemes and graphemes (Seymour et al., 2003; Lyytinen et al., 2015; Aro, 2017). Due to the transparency of Finnish orthography, most Finnish children learn to read accurately after few months of reading instruction in grade 1 (Lerkkanen et al., 2004), and by the time of grade 2 assessment [which was also the first assessment time point in the Catts et al. (2012) study], most are fluent readers and have a good command of reading comprehension skills (Lerkkanen et al., 2010). It is possible, then, that orthographic transparency could explain why there seem to be fewer cases of late-emerging RD among children learning to read in a context of transparent orthography as the differences in higher-level reading skills are visible already in grade 2. It is also likely that in a transparent orthography, it is possible to develop a resolving pathway more often despite early learning difficulties, as decoding task is cognitively less demanding. Additionally, Catts et al. (2012) followed the children until grade 8, whereas the present analysis extended only to grade 6. The longer gap between assessments may also increase the number of unstable RD cases. Finally, in our study, a somewhat stricter cut-off was used (10th percentile), while Catts et al. (2012) used the criterion of 1 standard deviation below the weighted sample mean (approximately 16th percentile).

Although the use of cut-offs is likely to lead to uncertainties in research findings because of measurement error (Francis et al., 2005; Branum-Martin et al., 2013), it is also a practical tool for the identification of children with RD. However, where and how we set the cut-off affects the identification of the RD groups and could possibly contribute to false impressions about the distinctness of the groups and about the risk of children for developing RD. Therefore, we need to find a way to use cut-offs without the evident problems accompanying them. One precaution against biased conclusions is the use of buffer zones around the single cut-offs (Shankweiler et al., 1999). The use of the buffer zone in the present study revealed a more complex picture than the one of the use of single cut-offs. For instance, the simulation without measurement error and without a buffer zone suggested that 4.38% of the children had late-emerging reading comprehension difficulties. However, when we use the buffer zone, we see that many of the children identified with late-emerging RD actually had borderline skills (lowest 10–25%) in reading fluency and/or reading comprehension already in grade 2. In other words, although many children passed the strict RD criterion, they nevertheless were still at the lower end of the skill distribution. Similarly, the simulation without the measurement error or buffer zone suggested that 2.48% of the children had resolving reading fluency difficulties, leading to the impression that these children were fluent readers in grade 6; in fact, most of them still scored in the borderline zone, just above the strict cut-off. The use of cut-off points is a practical tool for the identification of children with RD, but they can be problematic. Their use would be rational if we did not have a normal skill distribution, but this is not the case in reading achievement (Francis et al., 2005). Consequently, setting an arbitrary cut-off on the continuous distribution of reading achievement can lead to false or biased estimations of the prevalence of instability of the RD groups.

There are certain limitations in this study that need to be considered. First, we used only one measure for the assessment of reading comprehension in grades 2 and 6. Although we calculated the correction of attenuation in each grade in order to control measurement error, having more measures for the assessment of reading comprehension would have increased the strength of our model. Also, more measures and several time points would have allowed a more thorough and reliable assessment of reading comprehension.

In conclusion, this study shows that the use of measures with measurement error and the use of single cut-offs affect the longitudinal stability of RD identification across two time points. Comparing the prevalence of the groups arose from the use of single cut-off and those from the use of the buffer zone, it is evident that the use of single cut-off contributes to false impressions, such as how distinct the RD groups are. However, even after controlling for measurement error and using the buffer zone, our results suggest that RD are not stable over time for all children. Although many children manifest RD in the beginning of their school life and continue to have difficulties across grades, some children do not demonstrate difficulties until mid-primary school, and others may resolve their earlier difficulties by the end of primary school. Given that reading fluency and reading comprehension were not stable over time, the question arises about which additional factors affect the development of reading fluency and especially the development of reading comprehension, which was less stable. Further studies are needed to better understand the factors that could lead to late-emerging RD, either in reading fluency or in reading comprehension, as well as the factors that help children resolve their RD. Closer examination of the resolving cases could provide important information on the mechanisms that trigger protective factors. These insights could be used for the development of support systems and intervention programs, which will help children at risk for RD.

These results raise several clinical and practical implications. First, it seems that a change in the child’s RD status can occur both because of the effect of measurement error and because of the instability of RD identification. Consequently, because of the presence of measurement error in every assessment tool, it is not sufficient to diagnose RD based on only one assessment. Although some children may pass the strict cut-off, the results of the buffer zone show that they may still be in jeopardy for RD. Second, the use of a buffer zone along with continuous follow-ups of children’s reading development could facilitate more accurate identification of the children with RD. This is especially important in education systems in which access to remedial support or special needs interventions depends on an official diagnosis. Third, the accumulation of evidence for the instability of RD classification from this study and prior literature suggests a need for careful consideration of practices and permanency of diagnosing of RD. This is needed especially in cases where diagnostic practices deprive children with late-emerging RD of interventions or support and where individuals with resolving RD may continue to carry an inaccurate label or perception of one’s skills.

## Data Availability Statement

The datasets generated for this study are available on request to the corresponding author.

## Ethics Statement

The studies involving human participants were reviewed and approved by the Ethical Committee of the University of Jyväskylä. Written informed consent to participate in this study was provided by the participants’ legal guardian/next of kin.

## Author Contributions

MP and MT drafted the first version of the current manuscript. AT contributed to data analysis. M-KL and A-MP were responsible for data collection and commented on the manuscript. All authors contributed to the manuscript drafting, and read and approved the submitted version.

## Conflict of Interest

The authors declare that the research was conducted in the absence of any commercial or financial relationships that could be construed as a potential conflict of interest.

## References

[B1] American Psychiatric Association, (2013). *Diagnostic and Statistical Manual of Mental Disorders (DSM-5).* Washington, DC: American Psychiatric Association.

[B2] Aro M. (2017). “Learning to read finnish,” in *Reading Acquisition Across Languages and Writing Systems: An International Handbook* 1st Edn, eds Verhoeven L. T. W. Perfetti C. A. (Cambridge: Cambridge University Press), 416–436. 10.1017/9781316155752.017

[B3] Aro M. Wimmer H. (2003). Learning to read: English in comparison to six more regular orthographies. *Appl. Psycholinguist.* 24 621–635. 10.1017/s0142716403000316

[B4] Björn P. M. Aro M. T. Koponen T. K. Fuchs L. S. Fuchs D. H. (2016). The many faces of special education within RTI frameworks in the United States and Finland. *Learn. Disabil. Q.* 39 58–66. 10.1177/0731948715594787

[B5] Branum-Martin L. Fletcher J. M. Stuebing K. K. (2013). Classification and identification of reading and math disabilities: the special case of comorbidity. *J. Learn. Disabil.* 46 490–499. 10.1177/0022219412468767 23232442 PMC3836204

[B6] Catts H. W. Adlof S. M. Ellis Weismer S. (2006). Language deficits in poor comprehenders: a case for the simple view of reading. *J. Speech Lang. Hear. Res.* 49 278–293. 10.1037/0708-5591.49.2.125 16671844

[B7] Catts H. W. Compton D. Tomblin J. B. Bridges M. S. (2012). Prevalence and nature of late-emerging poor readers. *J. Educ. Psychol.* 104 166–181. 10.1037/a0025323 24273341 PMC3835401

[B8] Catts H. W. Hogan T. P. Fey M. E. (2003). Subgrouping poor readers on the basis of individual differences in reading-related abilities. *J. Learn. Disabil.* 36 151–164. 10.1177/002221940303600208 15493430 PMC2848965

[B9] Etmanskie J. M. Partanen M. Siegel L. S. (2016). A longitudinal examination of the persistence of late-emerging reading disabilities. *J. Learn. Disabil.* 49 21–35. 10.1177/0022219414522706 24596111

[B10] Florit E. Cain K. (2011). The simple view of reading: is it valid for different types of alphabetic orthographies? *Educ. Psychol. Rev.* 23 553–576. 10.1007/s10648-011-9175-6

[B11] Francis D. J. Fletcher J. M. Stuebing K. K. Lyon G. R. Shaywitz B. A. Shaywitz S. E. (2005). Psychometric approaches to the identification of LD IQ and achievement scores are not sufficient. *J. Learn. Disabil.* 38 98–108. 10.1177/00222194050380020101 15813593

[B12] García J. R. Cain K. (2014). Decoding and reading comprehension: a meta-analysis to identify which reader and assessment characteristics influence the strength of the relationship in English. *Rev. Educ. Res.* 84 74–111. 10.3102/0034654313499616

[B13] Gough P. B. Tunmer W. E. (1986). Decoding, reading, and reading disability. *Remed. Spec. Educ.* 7 6–10. 10.1177/074193258600700104

[B14] Holopainen L. K. Kiuru N. H. Mäkihonko M. K. Lerkkanen M. K. (2018). The role of part-time special education supporting students with reading and spelling difficulties from grade 1 to grade 2 in Finland. *Eur. J. Spec. Needs Educ.* 33 316–333. 10.1080/08856257.2017.1312798

[B15] Hoover W. A. Gough P. B. (1990). The simple view of reading. *Read. Writ.* 2 127–160. 10.1007/BF00401799

[B16] Hu L. Bentler P. M. (1999). Cutoff criteria for fit indexes in covariance structure analysis: conventional criteria versus new alternatives. *Struc. Equ. Model.* 6 1–55. 10.1080/10705519909540118

[B17] Landerl K. Wimmer H. (2008). Development of word reading fluency and spelling in a con-sistent orthography: an 8-year follow-up. *J. Educ. Psychol.* 100 150 10.1037/0022-0663.100.1.150

[B18] Landerl K. Wimmer H. Moser E. (1997). *Salzburger Lese- und rechtschreibtest [Salzburg Reading and Spelling Test].* Bern: Huber.

[B19] Leach J. M. Scarborough H. S. Rescorla L. (2003). Late-emerging reading disabilities. *J. Educ. Psychol.* 95 211–224. 10.1037/0022-0663.95.2.211

[B20] Lerkkanen M.-K. (2007). “The beginning phases of reading literacy instruction in Finland,” in *Finnish Reading Literacy. When Quality and Equity Meet*, eds Linnakylä P. Arffman I. (Jyväskylä: University of Jyväskylä), 155–174. 10.1108/s0735-004x(2010)0000023008

[B21] Lerkkanen M.-K. (2018). “The influence of instruction on reading motivation in Finland,” in *Reading achievement and Motivation in Boys and Girls: Field Studies and Methodological Approaches. Literacy Studies, 15*, eds Garcia P. O. Lind P. B. (Cham: Springer), 65–78. 10.1007/978-3-319-75948-7_4

[B22] Lerkkanen M.-K. (2019). “Early language and literacy development in the Finnish context,” in *The Sage Handbook of Developmental Psychology and Early Childhood Education*, eds Whitebread D. Grau V. Kumpulainen K. McClelland M. M. Perry N. E. Pino-Pasternak D. (London: Sage), 403–417. 10.4135/9781526470393.n23

[B23] Lerkkanen M.-K. Niemi P. Poikkeus A.-M. Poskiparta M. Siekkinen M. Nurmi J.-E. (2006). *The First Steps Study (Ongoing).* Finland: University of Jyväskylä.

[B24] Lerkkanen M.-K. Poikkeus A.-M. (2009). *Lausetasoinen luetun ymmärtäminen ja sujuvuus: TOSREC-testin adaptoitu ja lyhennetty versio: Alkuportaat-tutkimuksen testimateriaalia [Sentence reading efficiency and comprehension: Adapted and shortened version of the TOSREC test: Test material of the First Steps study] [Unpublished test material].* Finland: University of Jyväskylä.

[B25] Lerkkanen M.-K. Poikkeus A.-M. Ahonen T. Siekkinen M. Niemi P. Nurmi J.-E. (2010). Luku- ja kirjoitustaidon sekä motivaation kehitys esi- ja alkuopetusvuosina [The development of reading and spelling skills from kindergarten to Grade 2]. *Kasvatus* 41 116–128.

[B26] Lerkkanen M.-K. Rasku-Puttonen H. Aunola K. Nurmi J.-E. (2004). Predicting reading performance during the first and the second year of primary school. *Br. Educ. Res. J.* 30 67–92. 10.1080/01411920310001629974

[B27] Lindeman J. (1998). *ALLU–Ala-Asteen Lukutesti [ALLU–Reading Test for Primary School]*. Turku: University of Turku.

[B28] Lindeman J. (2000). *Ala-asteen lukutesti: Tekniset tiedot* ([2. p*.].). [ALLU Reading Test for Primary School: Technical Information].* Turku: University of Turku.

[B29] Lipka O. Lesaux N. Siegel L. (2006). Retrospective analyses of the reading development of Grade 4 students with reading disabilities: risk status and profiles over 5 years. *J. Learn. Disabil.* 39 364–378. 10.1177/00222194060390040901 16895160

[B30] Little R. J. A. (1988). A test of missing completely at random for multivariate data with missing values. *J. Am. Stat. Assoc.* 83 1198–1202. 10.1080/01621459.1988.10478722

[B31] Lyytinen H. Aro M. Richardson U. Erskine J. Banff A. Li U. H. (2015). “Reading skills, acquisition of: cultural, environmental, and developmental impediments,” in *International Encyclopedia of the Social & Behavioral Sciences*, 2nd Edn, ed. Wright J. D. (Amsterdam: Elsevier), 5–11. 10.1016/B978-0-08-097086-8.23111-5

[B32] Moll K. Kunze S. Neuhoff N. Bruder J. Schulte-Körne G. (2014). Specific learning disorder: prevalence and gender differences. *PLoS One* 9:e103537. 10.1371/journal.pone.0103537 25072465 PMC4114805

[B33] Nevala J. Lyytinen H. (2000). *Sanaketjutesti [Differentiate Word Test]*. Jyväskylä: Niilo Mäki Instituutti.

[B34] Pennington B. F. (2006). From single to multiple deficit models of developmental disorders. *Cognition* 101 385–413. 10.1016/j.cognition.2006.04.008 16844106

[B35] Perfetti C. A. (1985). *Reading Ability.* Oxford: Oxford University Press.

[B36] Pichler C. Wimmer L. (2006). Das salzburger Lesescreening 2-9. Handreichnung für Lehrerinnen und Lehrer. Based on Mayringer H. Wimmer H. (2003). Salzburger Lesescreening für die Klassenstufen 1-4 and Auer M. Gruber G. Mayringer H. Wimmer H. (2005). *Salzburger Lesescreening für die Klassenstufen.* 5–8.

[B37] Santos S. Cadime I. Viana F. L. Ribeiro I. (2019). Cross-lagged relations among linguistic skills in european portuguese: a longitudinal study. *Read. Res. Q.* 10.1002/rrq.261

[B38] Schatschneider C. Wagner R. K. Hart S. A. Tighe E. L. (2016). Using simulations to investigate the longitudinal stability of alternative schemes for classifying and identifying children with reading disabilities. *Sci. Stud. Read.* 20 34–48. 10.1080/10888438.2015.1107072 26834450 PMC4732731

[B39] Seymour P. H. K. Aro M. Erskine J. M. (2003). Foundation literacy acquisition in European orthographies. *Br. J. Psychol.* 94 143–174. 10.1348/000712603321661859 12803812

[B40] Shankweiler D. Lundquist E. Katz L. Stuebing K. K. Fletcher J. M. Brady S. (1999). Comprehension and decoding: patterns of association in children with reading difficulties. *Sci. Stud. Read.* 3 69–94. 10.1207/s1532799xssr0301_4

[B41] Snowling M. J. Hulme C. (2012). Annual Research Review: the nature and classification of reading disorders–a commentary on proposals for DSM-5. *J. Child Psychol. Psychiatry* 53 593–607. 10.1111/j.1469-7610.2011.02495.x 22141434 PMC3492851

[B42] Soodla P. Lerkkanen M. K. Niemi P. Kikas E. Silinskas G. Nurmi J. E. (2015). Does early reading instruction promote the rate of acquisition? A comparison of two transparent orthographies. *Learn. Instruc.* 38 14–23. 10.1016/j.learninstruc.2015.02.002

[B43] Statistics Finland (2005). *Erityisopetusta Saavien Määrän Kasvu Jatkui [Number of Students Receiving Special Education has Decreased].* Available at: http://www.stat.fi/til/erop/2004/erop_2004_2005-06-15_tie_001.html (accessed September 25, 2018).

[B44] Statistics Finland (2007). *Statistical Databases.* Available at: http://www.stat.fi/tup/tilastotietokannat/index_en.html (accessed September 25, 2018).

[B45] Torppa M. Eklund K. van Bergen E. Lyytinen H. (2015). Late-emerging and resolving dyslexia: a follow-up study from age 3 to 14. *J. Abnorm. Child Psychol.* 43 1389–1401. 10.1007/s10802-015-0003-1 25772426

[B46] Torppa M. Georgiou G. K. Lerkkanen M. K. Niemi P. Poikkeus A. M. Nurmi J. E. (2016). Examining the simple view of reading in a transparent orthography: a longitudinal study from kindergarten to grade 3. *Merrill-Palmer Q.* 62 179–206. 10.13110/merrpalmquar1982.62.2.0179

[B47] Torppa M. Tolvanen A. Poikkeus A. M. Eklund K. Lerkkanen M. K. Leskinen E. (2007). Reading development subtypes and their early characteristics. *Ann. Dyslexia* 57 3–32. 10.1007/s11881-007-0003-0 17849214

[B48] Tunmer W. E. Chapman J. W. (2012). The simple view of reading redux: vocabulary knowledge and the independent components hypothesis. *J. Learn. Disabil.* 45 453–466. 10.1177/0022219411432685 22293683

[B49] van Bergen E. van der Leij A. de Jong P. F. (2014). The intergenerational multiple deficit model and the case of dyslexia. *Front. Hum. Neurosci.* 8:346. 10.3389/fnhum.2014.00346 24920944 PMC4041008

[B50] Wagner R. K. Torgesen J. K. Rashotte C. A. Pearson N. A. (2009). *TOSREC: Test of Sentence Reading Efficiency and Comprehension.* Austin, TX: Pro-Ed.

