# Supplementary material for: Longitudinal Stability of Reading Difficulties: Examining the Effects of Measurement Error, Cut-Offs, and Buffer Zones in Identification
Source: Front Psychol. 2020 Jan 10;10:2841. doi: 10.3389/fpsyg.2019.02841 (PMC6965358; doi:10.3389/fpsyg.2019.02841)
Supplement: Supplementary file 1 [file Data_Sheet_1.pdf]

Supplementary Material

Appendix A

Mplus Input for Cross-Lagged Reading Model

```
TITLE: READING MODEL
DATA: FILE IS mplus.dat;
LISTWISE = ON;
VARIABLE: NAMES ARE childid cityid lukutaC8x difwC8c slsC8sum asiteksC8x
                    rtl2SC5 difwC5c rcsSC5 ly3C5c;
USEVARIABLES ARE lukutaC8x difwC8c slsC8sum asiteksC8x rtl2SC5 difwC5c rcsSC5
ly3C5c;
MISSING ARE ALL (-99);
ANALYSIS: ESTIMATOR=MLR;
          TYPE=GENERAL;
MODEL:   FlG2 BY RTL2SC5 DIFWC5C (1)
         RCSSC5 (2);
         FlG6 BY LUKUTAC8x DIFWC8C (1)
         SLSC8SUM (20);
         DIFWC5C WITH DIFWC8C;
         RTL2SC5 WITH LUKUTAC8x;
         RCSSC5 WITH SLSC8SUM;

!Kuder-Richardson reliability estimates for reading comprehension: ly3C5c: .80, asitetsC8x:
.74
! Variance ly3C5c=6.691
! Variance asitetsC8x=6.543
! Correction of attenuation (1-reliability)*variance
! For ly3C5c: (1-.80)*6.691=1.3382
! For asitetsC8x: (1-.74)*6.543=1.70118

         S1 BY LY3C5C@1; LY3C5C @1.3382;
         S1;
         S2 BY ASITEKSC8X@1; ASITEKSC8X @1.70118;
```

```
        S2;
        FlG6 ON FlG2 S1;
        FlG2 WITH S1;
        S2 ON S1 FlG2;
        FlG6 WITH S2;

OUTPUT: SAMP STAND MOD(4) RES;
```

Appendix B

Syntax Simulation without Measurement Error and Single Cut-off

Note: The same syntax is used for the simulation without measurement error and the buffer zone but instead of one cut-off, we have two.

```
DATA LIST FREE/NR.
BEGIN DATA.
 1 2 3 4 5 6 7 8 9 0 1 2 3 4 5 6 7 8 9 0 (At this point we enter 200,000 free data. Because it would take many pages to represent all 200,000, we have the first 20 as an example)
END DATA.

END DATA.
SET SEED=8949187.

*correlation is .549

COMPUTE COR1=NORMAL(1).
COMPUTE FLG2=SQRT(.549)*COR1+SQRT(1-.549)*NORMAL(1).
COMPUTE S1=SQRT(.549)*COR1+SQRT(1-.549)*NORMAL(1).
EXECUTE.

*residual correlation is .131
*residual variances are .250 for fluency and .593 for comprehension
*RESIDUALS

COMPUTE COR2=NORMAL(1).
COMPUTE RES_FL6=SQRT(.131)*COR2+SQRT(1-.131)*NORMAL(1).
COMPUTE RES_S2=SQRT(.131)*COR2+SQRT(1-.131)*NORMAL(1).
CORRELATIONS
    /VARIABLES=RES_FL6 RES_S2
    /PRINT=TWOTAIL NOSIG
    /MISSING=PAIRWISE.

COMPUTE FLG6=0.782*FLG2+0.139*S1+SQRT(0.250)*RES_FL6.
COMPUTE S2=0.568*S1+0.114*FLG2+SQRT(0.593)*RES_S2.
EXECUTE.

DESCRIPTIVES VARIABLES=FLG2 S1 FLG6 S2
  /STATISTICS=MEAN STDDEV MIN MAX.

SET DECIMAL=DOT.

WRITE OUTFILE='<DESTINATION TO SAVE NEW DATASET>' ENCODING='ASCII'/
    FLG2 FLG6 S1 S2 (4F12.3).
EXECUTE.

SORT CASES BY FLG2.
EXECUTE.
IF ($CASENUM LE 20000) FLG2_DIK=1.
```

```
IF ($CASENUM GT 20000) FLG2_DIK=0.
EXECUTE.
SORT CASES BY FLG6.
EXECUTE.
IF ($CASENUM LE 20000) FLG6_DIK=1.
IF ($CASENUM GT 20000) FLG6_DIK=0.
EXECUTE.
SORT CASES BY S1.
EXECUTE.
IF ($CASENUM LE 20000) S1_DIK=1.
IF ($CASENUM GT 20000) S1_DIK=0.
EXECUTE.
SORT CASES BY S2.
EXECUTE.
IF ($CASENUM LE 20000) S2_DIK=1.
IF ($CASENUM GT 20000) S2_DIK=0.
EXECUTE.

GENLOG FLG2_DIK S1_DIK FLG6_DIK S2_DIK
  /MODEL=POISSON
  /PRINT=FREQ RESID ADJRESID ZRESID DEV
  /PLOT=RESID(ADJRESID) NORMPROB(ADJRESID)
  /CRITERIA=CIN(95) ITERATE(20) CONVERGE(0.001) DELTA(.5)
  /DESIGN FLG2_DIK S1_DIK FLG6_DIK S2_DIK.
```

Appendix C

Syntax Simulation with Measurement Error and Single Cut-off

Note: The same syntax is used for the simulation with measurement error and the buffer zone but instead of one cut-off, we have two.

```
DATA LIST FREE/NR.
BEGIN DATA.
 1 2 3 4 5 6 7 8 9 0 1 2 3 4 5 6 7 8 9 0 (At this point we enter 200,000 free data. Because it would take many pages to represent all 200,000, we have the first 20 as an example)
END DATA.

SET SEED=37402897.

*correlation is .549

COMPUTE COR1=NORMAL(1).
COMPUTE FLG2=SQRT(.549)*COR1+SQRT(1-.549)*NORMAL(1).
COMPUTE S1=SQRT(.549)*COR1+SQRT(1-.549)*NORMAL(1).
EXECUTE.

*residual correlation is .131
* residual variances are .250 for fluency and .593 for comprehension
* RESIDUALS

COMPUTE COR2=NORMAL(1).
COMPUTE RES_FL6=SQRT(.131)*COR2+SQRT(1-.131)*NORMAL(1).
COMPUTE RES_S2=SQRT(.131)*COR2+SQRT(1-.131)*NORMAL(1).
CORRELATIONS
   /VARIABLES=RES_FL6 RES_S2
   /PRINT=TWOTAIL NOSIG
   /MISSING=PAIRWISE.

COMPUTE FLG6=.782*FLG2+.139*S1+SQRT(.250)*RES_FL6.
COMPUTE S2=.568*S1+.114*FLG2+SQRT(.593)*RES_S2.
EXECUTE.

DESCRIPTIVES VARIABLES=FLG2 S1 FLG6 S2
   /STATISTICS=MEAN STDDEV MIN MAX.

COMPUTE REC1=NORMAL(1).
COMPUTE REC2=NORMAL(1).
COMPUTE REC3=NORMAL(1).
EXECUTE.
COMPUTE RES1=SQRT(.073)*REC1+SQRT(1-.073)*NORMAL(1).
COMPUTE RES2=SQRT(.262)*REC2+SQRT(1-.262)*NORMAL(1).
COMPUTE RES3=SQRT(.264)*REC3+SQRT(1-.264)*NORMAL(1).

COMPUTE F21=.887*FLG2+SQRT(.213)*RES1.
```

```
COMPUTE F22=.597*FLG2+SQRT(.644)*RES2.
COMPUTE F23=.746*FLG2+SQRT(.444)*RES3.
COMPUTE S11=.894*S1+SQRT(.200)*NORMAL(1).
EXECUTE.
COMPUTE RES4=SQRT(.073)*REC1+SQRT(1-.073)*NORMAL(1).
COMPUTE RES5=SQRT(.262)*REC2+SQRT(1-.262)*NORMAL(1).
COMPUTE RES6=SQRT(.264)*REC3+SQRT(1-.264)*NORMAL(1).

COMPUTE F61=.852*FLG6+SQRT(.274)*RES4.
COMPUTE F62=.670*FLG6+SQRT(.551)*RES5.
COMPUTE F63=.736*FLG6+SQRT(.458)*RES6.
COMPUTE S21=.860*S2+SQRT(.260)*NORMAL(1).
EXECUTE.

COMPUTE RF2SUM=F21+F22+F23.
COMPUTE RF6SUM=F61+F62+F63.
COMPUTE LY3C5C=S11.
COMPUTE ASITEKSC8X=S21.
EXECUTE.

SET DECIMAL=DOT.

WRITE OUTFILE='<DESTINATION TO SAVE NEW DATASET>' ENCODING='ASCII'/
   F21 F22 F23 S11 F61 F62 F63 S21 (8F12.3).
EXECUTE.

SORT CASES BY RF2SUM.
EXECUTE.
IF ($CASENUM LE 20000) RF2_SDIK=1.
IF ($CASENUM GT 20000) RF2_SDIK=0.
EXECUTE.
SORT CASES BY RF6SUM.
EXECUTE.
IF ($CASENUM LE 20000) RF6_SDIK=1.
IF ($CASENUM GT 20000) RF6_SDIK=0.
EXECUTE.
SORT CASES BY LY3C5C.
EXECUTE.
IF ($CASENUM LE 20000) CO2_SDIK=1.
IF ($CASENUM GT 20000) CO2_SDIK=0.
EXECUTE.
SORT CASES BY ASITEKSC8X.
EXECUTE.
IF ($CASENUM LE 20000) CO6_SDIK=1.
IF ($CASENUM GT 20000) CO6_SDIK=0.
EXECUTE.

GENLOG RF2_SDIK CO2_SDIK RF6_SDIK CO6_SDIK
   /MODEL=POISSON
   /PRINT=FREQ RESID ADJRESID ZRESID DEV
```

```
/PLOT=RESID(ADJRESID) NORMPROB(ADJRESID)
/CRITERIA=CIN(95) ITERATE(20) CONVERGE(0.001) DELTA(.5)
/DESIGN RF2_SDIK CO2_SDIK RF6_SDIK CO6_SDIK.
```
